# Inflammatory phenotypes underlying uncontrolled childhood asthma despite inhaled corticosteroid treatment: rationale and design of the PACMAN2 study

**DOI:** 10.1186/1471-2431-13-94

**Published:** 2013-06-15

**Authors:** Susanne JH Vijverberg, Leo Koenderman, Francine C van Erp, Cornelis K van der Ent, Dirkje S Postma, Paul Brinkman, Peter J Sterk, Jan AM Raaijmakers, Anke-Hilse Maitland-van der Zee

**Affiliations:** 1 Division of Pharmacoepidemiology & Clinical Pharmacology, Utrecht Institute for Pharmaceutical Sciences (UIPS), Faculty of Science, Utrecht University, Universiteitsweg 99, Utrecht 3508 TB, the Netherlands; 2 Department of Respiratory Medicine, University Medical Centre Utrecht, Heidelberglaan 100, Utrecht 3584 CX, the Netherlands; 3 Department of Paediatric Respiratory Medicine, Wilhelmina Children's Hospital, University Medical Centre Utrecht, Lundlaan 6, Utrecht 3584 EA, the Netherlands; 4 Department of Pulmonology, Groningen Research Institute for Asthma and COPD, University of Groningen, University Medical Center Groningen, Hanzeplein 1, Groningen 9713 GZ, the Netherlands; 5 Department of Respiratory Medicine, Academic Medical Centre, University of Amsterdam, Meibergdreef 9, Amsterdam 1105 AZ, The Netherlands

**Keywords:** Asthma, Child, Phenotypes, Inflammation, Proteomics, Volatile organic compounds, Corticosteroids

## Abstract

**Background:**

The diagnosis of childhood asthma covers a broad spectrum of pathological mechanisms that can lead to similarly presenting clinical symptoms, but may nonetheless require different treatment approaches. Distinct underlying inflammatory patterns are thought to influence responsiveness to standard asthma medication.

**Methods/design:**

The purpose of the PACMAN2 study is to identify inflammatory phenotypes that can discriminate uncontrolled childhood asthma from controlled childhood asthma by measures in peripheral blood and exhaled air. PACMAN2 is a nested, case–control follow-up study to the ongoing pharmacy-based “Pharmacogenetics of Asthma medication in Children: Medication with Anti-inflammatory effects” (PACMAN) study. The original PACMAN cohort consists of children aged 4–12 years with reported use of asthma medication. The PACMAN2 study will be conducted within the larger PACMAN cohort, and will focus on detailed phenotyping of a subset of the PACMAN children. The selected participants will be invited to a follow-up visit in a clinical setting at least six months after their baseline visit based on their adherence to usage of inhaled corticosteroids, their asthma symptoms in the past year, and their age (≥ 8 years). During the follow-up visit, current and long-term asthma symptoms, medication use, environmental factors, medication adherence and levels of exhaled nitric oxide will be reassessed. The following measures will also be examined: pulmonary function, exhaled volatile organic compounds, as well as inflammatory markers in peripheral blood and blood plasma. Comparative analysis and cluster-analyses will be used to identify markers that differentiate children with uncontrolled asthma despite their use of inhaled corticosteroids (ICS) (cases) from children whose asthma is controlled by the use of ICS (controls).

**Discussion:**

Asthmatic children with distinct inflammatory phenotypes may respond differently to anti-inflammatory therapy. Therefore, by identifying inflammatory phenotypes in children with the PACMAN2 study, we may greatly impact future personalised treatment strategies, uncover new leads for therapeutic targets and improve the design of future clinical studies in the assessment of the efficacy of novel therapeutics.

## Background

Asthma is one of the most common chronic diseases in childhood [1]. It is increasingly recognized that asthma is not a homogeneous disease and that different pathological mechanisms can lead to the clinical expression of asthma [2]. Inhaled corticosteroids (ICS) have become the first-line controller therapy for asthma, and the standard treatment of persistent asthma is generally guided by symptom control [1]. Most children with persistent asthma symptoms will have a beneficial response to ICS. Nevertheless, there is large inter-individual variability [3] and a portion of children with asthma will remain uncontrolled despite intensive treatment with high dosages of inhaled corticosteroids and/or oral corticosteroids. Uncontrolled asthma leads to a lower quality of life, may induce lung damage, can cause life-threatening exacerbations and results in increased healthcare resources utilization and expenditures [4]. Therefore, it is essential to identify asthmatic children with a high risk of poor response to standard asthma medication at an early stage.

A poor treatment response to ICS can be caused by various factors, including poor therapy adherence, misdiagnosis or continued exposure to allergens [5]. In addition, biological factors, including genetic variations, seem to play an important role in inter-individual ICS responsiveness. A recent study by Tantisira *et al.* showed that asthma patients with a single-nucleotide polymorphism (SNP) in the gene *GLCCI1* have a worse response in lung function upon ICS treatment [6]. Furthermore, a SNP in the *FCER2* receptor gene has been associated with an increased risk of asthma-related hospital visits, uncontrolled asthma and higher daily steroid dosages [7,8]. Nevertheless, despite the progress in asthma pharmacogenetic research, only a small percentage of the variability in treatment response can currently be explained by variations in SNPs.

In addition to genetic polymorphisms, other biological factors such as distinct inflammatory patterns may influence ICS responsiveness. Inflammation in asthma is often described as ‘eosinophilic’, based upon the presence of primed eosinophils in the airways. However, it has been shown that airway inflammation in asthmatic patients may also occur in the absence of increased levels of eosinophils and in the presence or absence of neutrophilia [9,10]. Corticosteroids induce cell death in eosinophils, but can induce survival in other immune cells such as neutrophils [11]. Therefore, it is likely that asthmatic patients with distinct inflammatory phenotypes may vary in their response to corticosteroids. This has been confirmed by various studies showing that asthmatic patients with non-eosinophilic inflammation have a less beneficial response to corticosteroids when compared to those with eosinophilic inflammation [12]. In addition, a RCT carried out by Green and colleagues showed that titrating ICS treatment based on sputum eosinophilia led to better asthma control compared to titrating treatment based on standard asthma guidelines in adults without a significant difference in corticosteroid usage [13]. A cluster analysis by Haldar *et al.* showed that titrating treatment based on sputum eosinophilia to prevent exacerbations was superior in two clusters of patients (specifically refractory asthma) where markers of eosinophilic inflammation were discordant with the presence of asthma symptoms [2]. A recent RCT in severe asthmatic children found no differences in exacerbations or improvement of asthma control when treatment was adjusted based on sputum eosinophilia [14].

Various surrogate markers for airway inflammation have been described, including fraction of nitric oxide in exhaled breath (FeNO), volatile organic compounds (VOCs) in exhaled breath, sputum eosinophil counts and serum eosinophil cationic protein. Although these are, to a certain extent, applicable in clinical practice, few studies have assessed whether these markers are associated with ICS response in children [12]. In the PACMAN2 study we will focus on inflammatory phenotypes that may distinguish children who despite ICS use continue to suffer from asthma symptoms from children who are well controlled on ICS treatment. We aim to integrate clinical, proteomic, cellular and breath metabolomic data in order to more accurately define inflammatory mechanisms underlying asthma in children. PACMAN2 is an exploratory follow-up study of the ongoing Pharmacogenetics of Asthma medication in Children: Medication with Anti-inflammatory effects study (PACMAN) [15].

## Methods/design

### Study design

PACMAN2 is a nested case–control study within the observational pharmacy-based PACMAN cohort. PACMAN is an ongoing, cross-sectional study, including children aged 4–12 years with reported use of asthma medication. The inclusion criteria requires that they have had ≥3 prescriptions for Anatomical Therapeutic Chemical (ATC) code R03 medication in the past 2 years, including ≥1 prescription for R03 medication in the past 6 months. ATC code R03 medications are drugs prescribed for obstructive airway diseases and are comprised of short-acting beta-2 agonists, long-acting beta-2 agonists and inhaled corticosteroids (http://www.whocc.no/atc_ddd_index/?code=R03). Inclusion of children in the PACMAN cohort started in April 2009 and is still currently ongoing with over 990 children having been included thus far. Details of the study protocol of the PACMAN cohort study have been described elsewhere [15].

For the PACMAN2 study, specific subsets of children included in the PACMAN cohort (PACMAN) will be selected for a follow-up visit in a clinical setting. This visit will be planned for at least six months after the original baseline visit and will include the reassessment of asthma symptoms, medication use, adherence to ICS and levels of FeNO. Furthermore, additional measurements will be made including pulmonary function testing and the measurement of exhaled volatile organic compounds in exhaled breath and inflammatory markers in peripheral blood by immunophenotyping and proteomics approaches. Comparative analyses and cluster-analyses will be used to identify markers that discriminate children with uncontrolled asthma despite ICS use (cases) from children with controlled asthma on ICS (controls). Cases and controls will be classified according to current and long-term asthma control at the time of the follow-up study visit, as this is most likely to reflect their current disease state. Current, uncontrolled childhood asthma despite ICS usage will be the primary study endpoint.

**Figure 1:**
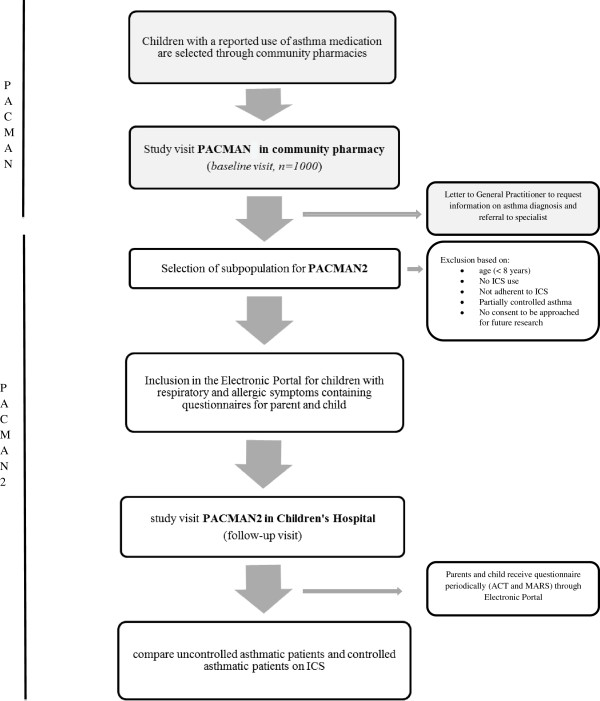
Flow chart data collection PACMAN cohort study and follow up.

Figure 1 presents a flowchart of the PACMAN study and the follow-up (PACMAN2).

### Selection of study subjects

Children in PACMAN2 will be selected from the PACMAN cohort based on the inclusion criteria shown in Table 1. In order to increase the probability of including both children with long-term well controlled and long-term poorly controlled asthma, children will be selected from the PACMAN cohort based on the following:

•The child’s asthma is classified as long-term controlled or long-term uncontrolled (see section below) at baseline, and

•The child is adhering to his/her inhaled corticosteroid regimen (Medication Adherence Rating Scale ≥ 21 [16]) at baseline.

**Table 1:** Inclusion criteria PACMAN2 study

**Long-term uncontrolled**	**Long-term controlled**
• Parental consent to be approached for future research	• Parental consent to be approached for future research
• 8 years of age or older	• 8 years of age or older
• Current ICS user	• Current ICS user
• Adherent to corticosteroids (MARS≥21)‡	• Adherent to corticosteroids (MARS≥21)‡
• Long-term uncontrolled in the past year‡	• Long-term controlled asthma in the past year‡
≥ 3 seasons in the past year in which symptoms were uncontrolled:	≥ 3 seasons in the past year in which symptoms were controlled:
o ≥ 3 of the following symptoms (daily or weekly)	o Following symptoms are not present or occur less than weekly:
▪ Daytime asthma symptoms (cough, wheeze, shortness of breath)	▪ Daytime asthma symptoms (cough, wheeze, shortness of breath)
▪ Nighttime asthma symptoms	▪ Nighttime asthma symptoms
▪ Limitations in daily activities	▪ Limitations in daily activities
▪ Rescue medication use	▪ Rescue medication use
	• No asthma-related ER visit in the past year‡
	• No OCS use in the past year‡

‡ Based on the retrospective questionnaire data obtained during the PACMAN pharmacy study visit (baseline). Current ICS use was confirmed during telephone contact prior to the follow up study visit. *ICS* Inhaled Corticosteroids, *MARS* Medication Adherence Report Scale, *OCS* Oral Corticosteroids, *ER* Emergency Room.

Current use of ICS will be checked when children are invited to participate in the follow-up study visit. Long-term and current asthma control will be reassessed during the follow-up study visit, as this may have changed over time. In the primary analysis we will compare children that are currently uncontrolled despite ICS usage at the time of the follow-up study to children whose asthma is controlled by the use of ICS at the time of the follow-up study.

### Current asthma control

Current asthma control will be assessed at both the baseline study visit and the follow-up study visit using the 6-item version of the Asthma Control Questionnaire (ACQ) (symptoms plus rescue medication use) [17]. An ACQ-score of < 0.75 will be considered as ‘well controlled asthma’, a score ≥ 0.75 will be considered ‘poorly controlled asthma’.

### Long-term asthma control

The definition of long-term asthma control is based on the guidelines of the Global Initiative for Asthma [1]. During the baseline study visit and the follow-up study visit, parents will be asked to score the presence of and frequency of the following asthma symptoms: 1) daytime symptoms (wheezing, coughing, and shortness of breath), 2) nighttime symptoms, 3) limitations in daily activities, and 4) use of rescue medication during all four seasons of the previous year. Using this data, the children’s asthma will be classified using the following definitions. Long-term, uncontrolled asthma is defined as ≥ 3 seasons of uncontrolled asthma in the past year with a season being considered uncontrolled when ≥ 3 asthma symptoms (daytime symptoms, daytime limitations, nighttime limitations or use of co-medication) occur on a daily or weekly basis [18]. Long-term controlled asthma is defined as ≥ 3 seasons of controlled asthma in the past year. A season is considered to be ‘controlled’ when asthma symptoms do not occur or occur less than weekly. In addition, children whose asthma is defined as long-term, controlled during the past year, but who reported the use of oral corticosteroids (OCS) or asthma-related ER visit(s) in the past year will be excluded.

### Data collection PACMAN2

Children and their parents selected from the PACMAN cohort will be invited to a study visit at the Wilhelmina Children’s Hospital. During this visit, data will be collected through the use of questionnaires and the measurements of lung function, exhaled breath, peripheral blood and blood plasma. Table 2 lists the instruments being used during the baseline visit (PACMAN) and the follow-up (PACMAN2).

**Table 2:** Instruments used during the baseline visit (PACMAN) and follow-up study visit (PACMAN2)

	**Baseline visit (PACMAN)**	**Follow-up (PACMAN2)**
Questionnaires		
	Questions on general health, allergies, asthma and respiratory symptoms	Questions on general health, allergies, asthma and respiratory symptoms
	Asthma control (ACQ-6) [19]	Asthma control (ACQ-6) [19]
	-	Childhood Asthma Control Test (c-ACT), Asthma Control Test (ACT) [20]§
	Questions on asthma control in the past 4 seasons [18]	Questions on asthma control in the past 4 seasons [18]
	Environmental factors (passive smoking, pets, living environment)	Environmental factors (passive smoking, pets, living environment)
		Active smoking is assessed in children > 12 years of age
	Beliefs about Medicines Questionnaire (BMQ) [21]	-
	Healthcare utilization for respiratory symptoms	Healthcare utilization for respiratory symptoms
	Exacerbations in the past year (ER visits/OCS usage)	Exacerbations in the past year (ER visits/OCS usage)
	Demographics	Demographics
	Current asthma medication use	Current asthma medication use
	Medication Adherence Rating Scale (MARS) [16]	Medication Adherence Rating Scale (MARS) [16] §
	-	General RAND questionnaire [22]
	-	Growth parameters, breast feeding and vaccination status
	-	Paediatric Asthma Quality of Life Questionnaire (PAQLQ) [23]§
	-	Paediatric and Adolescent Rhinoconjunctivitis Quality of Life Questionnaire (PRQLQ and AdolRQLQ) [24,25]§
	-	Allergic Rhinitis and its Impact on Asthma (ARIA) [26]§
	-	6-item Otitis Media Questionnaire (OM-6) [27]§
	-	Brouilette Score [28] §
	-	Food Allergy Quality of Life Questionnaire for children and teenagers (FAQLQ-CF and FAQLQ-TF) [29,30] §
	-	Self-Administered Eczema Area and Severity Index (SA-EASI) [31] §
	-	Children’s Dermatology Life Quality Index (CDLQI) and Infant’s Dermatitis Quality of Life Index Questionnaire [32] §
Inhalation technique		
	Inhalation technique (checklist)	*-*
Medication history		
	Medication history through pharmacy system	*-*
Lung function		
	Lung function testing and airway reversibility using hand-held diagnostic spirometer	Lung function testing and airway reversibility in a clinical setting by a trained lung function technician
Exhaled breath		
	Exhaled Nitric Oxide (FeNO) (Niox Mino)	Exhaled Nitric Oxide (FeNO) (Niox Mino)
	-	Volatile Organic Compounds
Saliva		
	Saliva sample (Oragene) for DNA	Saliva sample (Oragene) for DNA
Peripheral blood and plasma		
	-	Peripheral blood and plasma sample

§ In the Electronic Portal, parents and children are asked to answer ISAAC screening questions which aim to screen on the presence of atopic diseases. Based on their initial answers, the participants will then be prompted by the system to complete additional disease-topic specific questionnaires [33].

### Electronic portal

Before the scheduled follow-up visit, children and parents will be asked to complete an extensive online questionnaire regarding the child’s general health, respiratory symptoms, respiratory infections, hay fever, food allergy, eczema, as well as environmental factors. The online questionnaire is located in a patient portal (‘The Electronic Portal for children with respiratory and allergic symptoms’) developed by the Wilhelmina Children’s Hospital. The rationale and design of the Electronic Portal has been previously published [33].

In order to screen for the presence of atopic diseases, parents and children will be asked to answer screening questions based on the core questions of the International Study on Asthma and Allergies in Childhood (ISAAC) [34]. Based on their initial answers, the participants will then be prompted by the system to complete additional disease-topic specific questionnaires including the Asthma Control Test (ACT) [35], the Medication Adherence Rating Scale (MARS) [16] and the Paediatric Asthma Quality of Life Questionnaire (PAQLQ) [23]. The online questionnaire also contains questions on environmental factors such as tobacco smoke exposure, pet exposure and living environment. Additionally, after the follow-up study visit, parents and children will receive a short questionnaire (ACT and MARS) on the child’s current asthma symptoms and use of medication during each season (every three months).

### Additional questionnaire

In addition to the Electronic Portal questionnaires, parents and children will be asked to complete an additional, short questionnaire during their follow-up visit. This questionnaire will include the asthma control questionnaire (ACQ) [19] to assess asthma control in the previous week, questions regarding asthma symptoms during the previous seasons (to assess long-term asthma control), as well as questions about asthma-related health care utilization, recent severe exacerbations (OCS use, asthma-related ER visits and hospitalisation) and current asthma medication use.

### Lung function measurements and FeNO

Trained lung function technicians will perform spirometry and FeNO measurements. A single-breath, on-line measurement of FeNO will be carried out with a hand-held electrochemical analyser (NIOX Mino, Aerocrine, Solna, Sweden). FeNO is measured during the baseline visit in a similar manner. Lung function measurements will include: forced expiratory volume in one second (FEV_1_), forced vital capacity (FVC), and FEV_1_/FVC ratios before and after the inhalation of 800 μg salbutamol.

### Volatile organic compounds (VOCs) in exhaled breath

VOCs will be measured according to a validated method described previously [36]. In short, while wearing a nose-clip, children will be asked to breathe normally for 5 minutes through a three-way, non-rebreathing valve with a VOC filter (A2, North Safety, Middelburg, the Netherlands) at the inspiration port and a silica filter at the expiration port. Then, after taking a maximal deep inspiration, the child will be asked to exhale a single, vital capacity volume into a Tedlar bag connected to the expiration port and a silica reservoir to dry the exhaled air. The VOCs present in at least 500 ml of exhaled air in the Tedlar bag will be captured in Tenax GR Tubes (Interscience, Breda, The Netherlands) by using a peristaltic pump. The VOCs captured in the Tenax GR Tubes will be analysed with a validated panel of electronic noses (including carbon-poloymer, quartz microbalance metalloporphyrins, metal oxide sensors and ion mobility spectrometry) in the Department of Respiratory Medicine at the Academic Medical Centre in Amsterdam, The Netherlands [37].

### IgE levels, cytokines and chemokines in peripheral blood plasma

Levels of total and specific IgE against major allergens in plasma will be measured according the manufacturer’s instructions using the Phadia ImmunoCAP system (UniCAP, Pharmacia, Sweden). These tests allow quantitative measurements (in kilo antibody units per litre; kU/l) of total IgE antibodies and specific IgE antibodies against common respiratory allergens. A concentration of specific IgE of 0.35 kU/l will be used as a cut-off value for a positive test result. In addition, cytokines and chemokines will be measured using multiplex immunoassay technology.

### Immunophenotyping of peripheral blood cells

Expression of a wide range of surface markers on peripheral blood cells will be determined using multi-colour flow cytometry (Gallios, Beckman Coulter, Woerden, The Netherlands). Venous blood will be collected in sterile collection tubes containing sodium heparin as anticoagulant. Shifts in activation profiles of inflammatory cells will be assessed. The function of inflammatory cells is associated (in part) with the activation status of the cells’ receptors, which can be modulated upon priming with inflammatory mediators, such as cytokines, chemokines and bacterial products. The expression of surface markers on distinct types of inflammatory cells will also be measured.

### Proteomic profiling of peripheral blood granulocytes in vitro treated +/− dexamethasone

The *in vitro* effect of corticosteroids on the protein expression of peripheral blood granulocytes will be assessed using fluorescence 2-dimensional difference (2D) gel electrophoresis [38]. In brief, granulocytes will be isolated from whole blood anticoagulated with sodium-heparin using Ficoll-Paque. Granuloytes (5.10^6^/mL) in incubation buffer will be treated *in vitro* with dexamethasone (10^-6^ M) or sham-treated with phosphate buffered saline for 15 minutes at 37°C. Subsequently, the cells will be stimulated with TNFα (100U/mL) for 3 hours. Cells will be lysed in lysis buffer complemented with protease inhibitors, and proteins will be precipitated with 80% acetone and dissolved in 2D labelling buffer. 2D-DIGE technology will be used to analyse the proteomics samples and the differential protein expression after dexamethasone treatment. Spot detection will be performed with DeCyder 7.0 Difference in-gel Analysis software (GE Healthcare, Uppsala, Sweden) and gel images will be matched using DeCyder 7.0 Biological Variation Analysis software (GE Healthcare, Uppsala, Sweden).

### Study endpoints

We will assess whether a (combination of) inflammatory marker(s) is (/are) associated with:

current, uncontrolled childhood asthma despite ICS usage (primary study endpoint).

long-term, uncontrolled childhood asthma despite ICS usage (secondary study endpoint).

### Statistical analyses

Statistical analyses to compare controlled and uncontrolled asthma patients with respect to lung function, FeNO, total and specific IgE levels and flow cytometry data (surface and internal markers on immune cells) will be performed using independent sample *t* tests or one-way ANOVA with Dunnett’s multiple comparison test for variables with a normal distribution, and Mann–Whitney and Kruskall Wallis tests for variables with non-normal distributions. In addition, unbiased cluster analysis will be used to assess patterns of inflammation. Statistical analysis of 2D-DIGE spot intensity will be performed using DeCyder 7.0 Extended data analysis software (GE Healthcare, Uppsala, Sweden) as described previously [38]. Since we aim to identify a fingerprint of markers that are differentially expressed, we will further explore the data using principal component analysis and other clustering-analyses on the entire data set (including VOCs data) with adequate (cross-)validation according to recent recommendation in order to limit false-discovery [39,40]. Sensitivity analyses will be performed on the variables current adherence to corticosteroid treatment and continued exposure to environmental factors (pet exposure, passive/active smoking).

### Sample size calculation

Data concerning inflammatory markers in peripheral blood for long-term, uncontrolled asthma in paediatric asthma patients are lacking in the current literature; therefore, we are not able to perform a sample size calculation. However, it has been shown that proteomic approaches can distinguish protein expression profiles of peripheral blood cells in studies with small numbers of asthmatic patients and controls (n≥6) [41]. Therefore, the following sampling approach has been selected.

Based on a preliminary analysis of 744 children included in the PACMAN cohort, we found that 86.4% of the children use ICS and 60.2% are adherent to ICS treatment. When we assessed long-term asthma control at baseline, 33.4% of the children were long-term well controlled, 53.3% of the children were long-term partially controlled and 13.3% of the children were long-term poorly controlled. We expect that approximately 5.3% (n=53) of the children in the final PACMAN population (n=1000) will fulfil all the inclusion criteria for the uncontrolled (adherent) asthma patients and 12.5% (n=125) will fulfil all the inclusion criteria for the controlled (adherent) asthma patients. We will therefore invite all of the children that fulfil the inclusion criteria of ‘uncontrolled asthma patients’, as well as an equal number of controlled asthma patients.

### Ethics

Only children whose parents consented to being approached for future research studies during their PACMAN study visit in the pharmacy will be invited to participate in the PACMAN2 study. A written informed consent will be obtained from the parents and from children who are ≥12 years. The Medical Ethics Committee of the University Medical Centre Utrecht approved this study.

## Discussion

The PACMAN2 study represents an in-depth approach to the assessment of inflammatory phenotypes of steroid-treated, asthmatic children. It is our aim to integrate clinical data with inflammatory patterns in exhaled breath and peripheral blood in order to accurately assess paediatric asthma phenotypes related to asthma control, thereby gaining more insight into the underlying inflammatory mechanisms.

Many recent studies have focused on improving response to asthma medication using inflammatory or genetic markers; so far, success has been limited. Asthma is a heterogeneous disease and individual corticosteroid responsiveness is mostly likely to be the sum of various factors, including adherence to treatment, absence or presence of co-morbidities, exposure to allergens, genetic variations in therapeutic targets or pathways, as well as inflammatory patterns that may be intrinsically more or less sensitive to corticosteroid treatment [5]. All these different factors have to be taken into account for optimal guidance of individual treatment. Therefore, accurate phenotyping of children with asthma is paramount.

In 2009, the PACMAN cohort study was started in order to assess the effectiveness of asthma medication in children and the influence of genetic factors [15]. This resulted in a unique, pharmacy-based paediatric cohort representing a cross-section of children who use asthma medication on a regular basis. The asthma phenotypes ranged from controlled to uncontrolled asthma and from patients with mild disease primarily treated by general practitioners to patients with moderate to severe disease receiving specialized care from paediatricians or paediatric pulmonologists. The added value of the PACMAN cohort over that of other existing population-based asthma cohorts is its primary focus on medication use, in contrast to other paediatric asthma cohorts that have mainly concentrated on determinants of asthma susceptibility or respiratory symptoms [42-44]. Furthermore, large studies that have assessed treatment effectiveness in asthmatic children, such as the Childhood Asthma Management Program (CAMP) [45] or the BREATHE study [46] have not taken underlying inflammatory patterns into account.

Defining appropriate therapy responses for asthma is a complex issue because of the heterogeneity of the disease. Various outcomes have been used to study the effectiveness of asthma therapies, for example improvement in lung function, symptoms scores or exacerbations (frequently defined by asthma-related hospital admissions, ER visits and/or oral corticosteroid use). Yet it is important to realize that predictors of treatment response depend upon the chosen definition of outcome variables [47,48]. Work by Haldar *et al.* showed that distinct clusters of adult asthmatics can be identified when studying two distinct dimensions of disease, i.e. asthma symptoms and eosinophilic inflammation [2]. These clusters can be concordant (asthmatic symptoms and measures of inflammation correlate) or discordant (asthmatic symptoms and measures of inflammation do not correlate). Others have described distinct inflammatory phenotypes based on sputum profiles of asthmatics [9,10], indicating that defining outcomes solely on the presence or absence of symptoms or solely on markers of (eosinophilic) inflammation may only be informative for a subgroup of the total patient population. Therefore, in PACMAN2, we aim to collect data on symptoms as well as on inflammation markers.

During the first phase of the PACMAN study we obtained saliva samples for DNA extraction from our participants. To date, enough saliva has been collected for sufficient DNA isolation in 74% of the children (550/744). Recently, we replicated the genetic association identified by Tantisira *et al.* between the *FCER2* T22026 gene variant and treatment response in asthmatic children and showed that this SNP is associated with an increased risk of asthma-related hospital visits in our population [7,8]. The follow-up of specific subsets in PACMAN2 will give us the opportunity to identify new pharmacogenetic targets using proteomic and cellular profiling strategies and to validate these in the PACMAN cohort.

Markers in exhaled breath, FeNO and VOCs, will also be measured in an attempt to further elucidate their applicability in identifying asthma phenotypes in children; however the direct correlation between FeNO and airway inflammation remains unclear. FeNO is thought to be a marker of eosinophilic airway inflammation, but various other factors including steroid use and atopy seem to significantly influence FeNO levels [49]. Several studies have reported that high levels of FeNO in asthmatics are associated with a better response to ICS [50-52].

Measuring patterns of VOCs in exhaled breath is a relatively novel metabolomic approach to study molecular signatures of respiratory disease. Exhaled breath contains a complex mixture of up to thousands of VOCs. These compounds are produced due to metabolic processes and the concentrations are likely to be influenced by the presence of airway inflammation. An electronic nose assesses the spectrum of volatiles present in exhaled breath without determining the individual molecular components [53,54]. Previous studies have shown that measurements of patterns of VOCs (‘breathprints’) using an electronic nose could discriminate adults with asthma from non-asthmatic controls [55] and asthmatic patients from COPD patients [36,56]. Furthermore, a recent study showed that breathprints of COPD patients with mild disease correlate well with the activation status of eosinophils and neutrophils in induced sputum samples of these patients [57], suggesting that the electronic nose might be promising non-invasive diagnostic tool to assess ongoing airway inflammation.

An important strength of PACMAN2 is the extensive phenotyping of steroid-treated asthmatic children and the follow-up over time. We will reassess asthma symptoms, medication use, adherence and FeNO levels in children who were long-term, uncontrolled or controlled at the baseline visit. Nonetheless, due to the fact that the definition of long-term asthma control will be based upon retrospective questionnaire data, with parents and children being asked to answer questions about asthma symptoms over the past four seasons, recall bias may occur leading to an over- or underestimation of the symptoms, constituting a potential limitation of our approach. Even so, long-term asthma control will likely provide additional information compared to current asthma control solely and findings from PACMAN2 will provide a better understanding of inflammatory phenotypes that may underlie uncontrolled asthma in inhaled steroid-treated children.

Childhood asthma affects millions of children worldwide and it is the leading cause of emergency room visits and hospitalizations in children, resulting in increased healthcare resources utilization and expenditures, and ultimately, costs to society. A substantial proportion of these asthma-related hospital visits occur despite high dosages of corticosteroid treatment. The identification of (inflammatory) phenotypes that reflect the pathobiological mechanisms underlying poor corticosteroid response may be of great importance in identifying high-risk patients at an early stage. Results from the PACMAN2 study might eventually lead to a more individualized treatment approach for asthmatic children, as well as to the discovery of new leads for innovative therapeutic strategies.

## Abbreviations

ACQ: Asthma control questionnaire; ACT: Asthma control test; CAMP: Childhood asthma management program; ER: Emergency room; FeNO: Fraction of exhaled nitric oxide; FEV1: Forced expiratory volume in one second; FVC: Forced vital capacity; ISAAC: International study on asthma and allergies in childhood; ICS: Inhaled corticosteroids; MARS: Medication adherence rating scale; OCS: Oral corticosteroids; PAQLQ: Paediatric asthma quality of life questionnaire; RAND-GHRI: RAND general health rating index; SNP: Single nucleotide polymorphism; VOC: Volatile organic compounds; 2D-gel: 2-Dimensional difference gel.

## Competing interests

Susanne J.H. Vijverberg has been paid by an unrestricted grant from GlaxoSmithKline (GSK). Jan A. M. Raaijmakers is a part-time professor at the Utrecht University and Vice-president External Scientific Collaborations for GSK in Europe, and holds stock in GSK. Anke-Hilse Maitland-van der Zee received an unrestricted grant from GSK. Cornelis K. van der Ent received unrestricted grants from GSK and Grunenthal. Dirkje S. Postma has received fees for consultancy work or unrestricted grants from AstraZeneca, Boehringer Ingelheim, Chiesi, GSK, Nycomed, TEVA. Peter J. Sterk has received a university grant from the University of Amsterdam, from the Innovative Medicines Initiative and from GSK. Furthermore, the Department of Pharmacoepidemiology and Clinical Pharmacology, Utrecht Institute for Pharmaceutical Sciences, employing authors Susanne J.H. Vijverberg, Jan A.M. Raaijmakers, and Anke-Hilse Maitland-van der Zee, has received unrestricted research funding from the Netherlands Organisation for Health Research and Development (ZonMW), the Dutch Health Care Insurance Board (CVZ), the Royal Dutch Pharmacists Association (KNMP), the private-public funded Top Institute Pharma (http://www.tipharma.nl, includes co-funding from universities, government, and industry), the EU Innovative Medicines Initiative (IMI), EU 7th Framework Program (FP7), the Dutch Medicines Evaluation Board, the Dutch Ministry of Health and industry (including GSK, Pfizer, and others). Leo Koenderman, Francine C. van Erp and Paul Brinkman declare to have no competing interests.

## Authors’ contributions

SV, AHMvdZ, JR, LK, DP and CvdE conceived the design of the study. SV drafted the manuscript. FvE and CvdE designed and coordinated Portal and the implementation in PACMAN2. PB and PS developed the VOCs analysis and implementation in PACMAN2. All authors read and approved the final manuscript.

## Pre-publication history

The pre-publication history for this paper can be accessed here:

http://www.biomedcentral.com/1471-2431/13/94/prepub
